# The Linked Response: Lessons Emerging from Integration of HIV and Reproductive Health Services in Cambodia

**DOI:** 10.1155/2013/504792

**Published:** 2013-01-31

**Authors:** Joanna White, Thérèse Delvaux, Chhorvann Chhea, Sarun Saramony, Vichea Ouk, Vonthanak Saphonn

**Affiliations:** ^1^Department of Public Health, Institute of Tropical Medicine, 155, Nationalestraat, 2000 Antwerp, Belgium; ^2^Centre for Research in Anthropology (CRIA), Lisbon University Institute, (IUL), 1649-026 Lisbon, Portugal; ^3^Surveillance Unit, National Center for HIV/AIDS, Dermatology and STD, 855 Phnom Penh, Cambodia; ^4^Resarch Unit, National Center for HIV/AIDS, Dermatology and STD, 855 Phnom Penh, Cambodia; ^5^Technical Bureau, National Center for HIV/AIDS, Dermatology and STD, 855 Phnom Penh, Cambodia

## Abstract

A qualitative assessment was made of service provider and user perceptions of the quality of integrated reproductive health services established through a pilot intervention in Cambodia. The intervention aimed to promote pregnant women's HIV testing and general utilization of reproductive health facilities as well as improve the follow-up of HIV-positive women and exposed infants through strengthened referral and operational linkages amongst health facilities/services and community-based support interventions for PLHIV. The study was conducted in one operational district where the intervention was piloted and for comparative purposes in a district where integrated services had yet to be implemented. Service providers in the pilot district reported improved collaboration and coordination of services, more effective referral, and the positive impact of improved proximity of HIV testing through integrated local level facilities. Community-based support teams for PLHIV embraced their expanded role, were valued by families receiving their assistance, and were understood to have had an important role in referral, PMTCT follow-up and countering PLHIV stigmatization; findings which underscore the potential role of community support in integrated service provision. Challenges identified included stigmatization of PLHIV by health staff at district hospital level and a lack of confidence amongst non-specialized health staff when managing deliveries by HIV-positive women, partly due to fear of HIV transmission.

## 1. Introduction

Over the last decade, the importance of linking sexual and reproductive health and HIV policies, systems, and services has been increasingly recognized. Several studies have shown how such linkages might be beneficial for both HIV and reproductive health (RH) programmes [1, 2]. More specifically, efforts have been deployed to integrate HIV testing, care, and, more recently, treatment within antenatal and delivery care services as a strategy to prevent mother-to-child transmission of HIV (PMTCT; [3]). A systematic review concluded that limited, nongeneralizable evidence supports the effectiveness of integrated PMTCT programmes versus non- or partially integrated services [4]. Limited evidence also suggests that PMTCT integration may improve the overall quality of antenatal (ANC) and delivery care services and female attendance at ANC services [4–9], although causal relationships are hard to ascribe [10]. Conversely, however, some commentators have noted that the integration of PMTCT might decrease quality of care and have potential negative effects on the workload of ANC service providers, risks which should be taken into account before integrating additional activities [7, 9]. Further, while in theory the linking of sexual and reproductive health and HIV can act as a modality of stigma reduction, this potential has not yet been clearly demonstrated and more work in this area by programme managers has been advocated [2]. While in many countries, including Cambodia, community-based organisations (CBOs) play an important role in PMTCT at local level, and renewed interest has been expressed in the potential role of community-based interventions in the provision of a continuum of care for maternal, newborn, and child survival [11], there is little evidence concerning the role and contribution of CBOs in the provision of integrated PMTCT and reproductive health services [12, 13].

The Linked Response (LR) approach was piloted in Cambodia with the aim of integrating HIV and RH services in order to ensure pregnant women were referred to health facilities to receive HIV tests and other support and to facilitate the follow-up of HIV-positive women and exposed infants [14]. While Cambodia had made significant progress in reducing HIV prevalence (from 2.4% in 1999 to 1.1% in 2006), halting the spread of HIV through concerted prevention strategies and universal antiretroviral therapy (ART) coverage, and improving the quality of life for people living with HIV (PLHIV) through government-run programmes [15], there were concerns regarding the number of new infections passed from mothers to newborns [16, 17]. When the LR was introduced in 2008, despite a substantial increase only 29% of pregnant women were being tested for HIV and 27% of identified HIV-positive pregnant women had been provided with ART regimens for PMTCT [15]. Although PMTCT services had been scaled up to many operational districts (ODs), in most cases these were only available at the district hospital. Poor referral mechanisms, decentralized services, lack of geographical access and transportation difficulties were identified as barriers to pregnant women testing for HIV and factors contributing to HIV-positive pregnant women and exposed infants being lost to follow-up before being provided with ART [16].

The LR was piloted in five operational districts from 2008 to 2009 and has been described in detail elsewhere [18]. The pilot operated through a “hub,” located at district referral hospital level, providing a full package of services, including treatment of opportunistic infections (OIs) and ART; “satellite” sites, providing a comprehensive package of activities including voluntary counselling and testing (VCT) and PMTCT services; “linked” health centres, offering a minimum package of activities including ANC, with integrated HIV counselling and testing (blood drawn and samples transferred to “satellites” for laboratory testing) as well as delivery care. District services were further coordinated by bringing together operational managers—HIV/AIDS and Maternal and Child Health Coordinators—and health facility staff through systematic service planning, implementation, and monitoring activities. CBOs, already providing care and support to PLHIV through Home-Based Care (HBC) teams, were asked to broaden their scope of work to include encouraging all pregnant women to test for HIV, raising awareness of PMTCT and RH and broader community sensitization activities.

Quantitative routine data from one of the pilot LR sites collected before, during, and after the intervention (in 2007, 2008, and 2009, resp.) revealed a dramatic increase in the proportion of pregnant women testing for HIV: from 3% in 2007 to 55% in 2008 and 80% in 2009. This contrasted favourably with routine data from a comparable OD which was without LR but with a PMTCT intervention run by a nongovernmental organisation (NGO), where testing increased from 3% in 2007 to 10% in 2008 and 34% in 2009, based on estimates of the total expected number of pregnant women in the respective ODs [18]. The objective of the current study was to qualitatively assess both service provider and user perceptions of the quality of the various services which constituted the LR in one district where the intervention was piloted and a second comparable OD where the LR had not yet been implemented.

## 2. Materials and Methods

The research team was composed of one study manager, one field coordinator, and four field researchers (all Cambodian), supported by two international research advisors. None of the researchers had been involved in managing or providing the health services which were the focus of the study. The field researchers received two days training prior to conducting the fieldwork which focused on the aims and content of the LR and research techniques and ethics. The study protocol was approved by the National Ethical Committee for Health Research (NECHR) in Cambodia, the Institutional Review Board at the Institute of Tropical Medicine (ITM), and the Ethical Committee at the University of Antwerp in Belgium.

The assessment was conducted in a district where the LR was introduced in 2008 (OD1) and a second OD where the LR only became operational in 2011 (OD2). Both districts were comparable in terms of geographical situation, rural context, and major health indicators. A similar methodological approach was employed in both of the districts studied, except that interviews and focus group discussions in OD2 did not include specific questions on the LR, but focused on study participants' experience with ANC, PMTCT, delivery, and follow-up services in the period since 2008, up to the introduction of the LR. The study was conducted over an eight day period in December 2010 and followed a purposive sampling approach.

### 2.1. In-Depth Interviews

A total of eight and two interviews with HIV-positive women were conducted in OD1 and OD2, respectively. In both ODs HIV-positive women interviewees were selected from a list held by the HBC teams; HBC team representatives then approached these women individually and invited them to participate. In OD1 one-to-one semistructured in-depth interviews (IDIs) were conducted with eight HIV-positive women who had given birth since the LR was operational. In total, ten women were approached but two declined to participate in the study (one citing lack of free time and the other her unwillingness to be interviewed). Each interview followed a questionnaire and explored ANC attendance during most recent pregnancy, HIV testing, disclosure of status, decision about pregnancy outcome, access to PMTCT, details about most recent delivery, role of partner and family in HIV testing, disclosure and treatment adherence, general support provided by health services and HBC teams, and respondents' satisfaction with services. IDIs exploring similar topics were also carried out with the partner, or close family member, of five of the eight HIV-positive women interviewed. Respondents were asked if they were willing for their partner or a family member to be interviewed, and if so, to introduce the research team to this individual. Some interviewees were not willing to include their partners/family members in the study, hence the lower number of partner/family respondents than female PLHIV interviewees in the study. Partners/family member interviewees of HIV-positive women were selected by the women themselves.

Given that, according to existing data, HIV prevalence in OD2 was comparable to that in OD1 at the time of the study, a full, comparative study was originally planned for this second district. However low numbers of women were found to be registered with health services as HIV-positive in OD2 at the time of the study. This may have been due first to the lack of effective implementation of PMTCT services at that stage since the LR was not yet scaled up in that district or secondly, because of women's unwillingness to be contacted and identified in an OD where the CBOs present were not yet involved in PMTCT services. Therefore it was not possible to identify a large sample who had delivered within the same period as those interviewed in OD1. As a result the inclusion criteria were expanded in OD2 to include pregnant women. Two PLHIV were interviewed: one woman who had an infant of around three months of age and one pregnant woman (a third HIV-positive woman was invited to participate in the study but refused, citing fear of disclosure of her status to the research team). One IDI was conducted with a partner/family member of the PLHIV interviewed, again chosen by the woman herself.

An IDI was also conducted with the AIDS Coordinator in OD1 (also Deputy Director of district health services), which explored the current functioning of PMTCT services, the impact of LR, current challenges, and suggestions for improvement. In OD2 an IDI was conducted with the AIDS Coordinator, who prior to the operationalization of LR in early 2011 had held the position of Mother and Child Health Program Manager. This interview contrasted the historical functioning of PMTCT services and the new system introduced by the LR and current challenges and suggestions for improvement.

### 2.2. Focus Group Discussions

To gain information from the broader population, two focus group discussions (FGDs) were held with women in OD1 who were not known to be HIV-positive and had given birth since the LR was operational. These discussions followed thematic guidelines, and included knowledge and understanding of HIV and PMTCT, awareness and utilization of RH services, and engagement with the local HIV testing process. Participants were also asked about their satisfaction with the quality of specific RH services provided by local facilities. Two groups of five women participated in the FGDs which took place in two community settings within the OD, up to ten kilometers from the district town. Similarly, two FGDs were held with women from the general population in OD2 who had given birth since 2008. Again, these women, who were not known to be HIV-positive, were drawn from two different communities within the OD. One FGD included four women and the other five women and both took place in local settings. All FGD participants were randomly selected from lists held by village leaders and at local health facilities.

To elicit the opinions and experiences of health staff working at different levels of service provision in OD1 one FGD was held with five health staff working in a range of roles at the “hub” (the district referral hospital), including PMTCT, maternal health and laboratory work, and a second FGD was held with five staff working at either “satellite” or “linked” health centres. The discussion included observed changes in the functioning of HIV testing and PMTCT services since the introduction of the LR, working partnerships, constraints to service provision, and the relationship with service users. A further FGD was held with five representatives of HBC teams, who are employed by a range of NGOs but, in line with the LR approach, work closely with the government health facilities. The discussion included views on the coverage and quality of the services provided through HBC teams, relationship with beneficiaries, and working partnerships with public health service providers. In OD2 one FGD was held with five staff working at the referral hospital, a second FGD was held with four midwives working at “satellite” or “linked” health centres and a further FGD was held with HBC representatives, though these were not yet involved in PMTCT. Health staff and HBC representatives chosen to attend FGDs were purposively selected by the research team from staff lists, with the aim of including individuals with diverse responsibilities and working experience.

IDI and FGD guidelines were pretested and modified as needed by the research team prior to the study formally commencing. A private place was used for all FGDs and IDIs and all participants provided full, written informed consent in Khmer prior to any data collection.

All interviews and FGDs were conducted in Khmer and digitally recorded. The recordings were transcribed and then translated into English using Microsoft Word (Version 2007) and were analysed by one of the international research advisors, in close consultation with the study manager, using a content analysis approach, following an inductive coding method. 

### 2.3. Study Limitations

The researchers are aware that the non-random selection of HIV-positive interviewees from lists held by HBC teams may have led to some biases in sampling and, subsequently, the data collected. By definition, only HIV-positive women registered and actively engaged with local services were included; the study did not include those who may have been lost to follow-up or were unwilling to engage with services, hence the data presented may not reflect the full range of women's experience. However in Cambodia, given the very high coverage of ART (reaching more than 95% of those in need), the majority of the HIV-positive population is likely to be registered. Nevertheless, a number of HIV-positive women in the OD2 may have not been registered because of they had not been tested for HIV and were not aware of their HIV status. Reaching such individuals was beyond the control of the study.

## 3. Results and Discussion

### 3.1. Positive Impacts of Integrated Approach on Provider Collaboration and Service Provision

The introduction of the LR was considered a positive development by the HIV/AIDS Coordinator and all service provider FGD participants in OD1. A member of PMTCT staff at the “hub” summarized the positive impacts of the integrated approach as follows:

*“We get a lot of advantages from this programme (LR). First, we gain more clients for HIV testing among pregnant women. So as a result of establishing this programme, first we reduce HIV infection, prevent transmission from mother to child, and sometimes reduce the mortality rate of mother and children. Because if they are HIV-positive, the doctors will know about how many CD4 they have, and how OI services can help them. Their name is noted in order to know about their delivery date and give them information to make them come to get ANC. We didn't have any of this before”.*

Health staff who attended FGDs also reported improved collaboration amongst the various departments and individuals responsible for OIs, ANC, and PMTCT as a result of the intervention. A member of maternal health staff working at the “hub” emphasised the improved referral and follow-up system, for example:

*“For the previous PMTCT programme, if we had an HIV-positive patient, we didn't keep any document or put any code number for them. But since we have the Linked Response… if we find a positive case we will refer them to OI/ART and we keep maternity records. We educate them to get ANC check-ups. The difference is that we register them properly. In the past, we didn't do that”.*

Similarly, another member of hospital staff described:

*“Since we have Linked Response, it's easier to refer patients from place to place and keep track of their condition… Before we didn't know clearly why patients were referred to us, but now we all know… what should be done if someone is found positive, and if a patient is referred to us… All of this is the part of the Linked Response”.*

“Linked” health centre staff particularly emphasised the importance of the improved proximity of HIV testing facilities provided through the LR:

*“When the blood testing service is close by then they come to test… Before we (the testing service) was far away so when we told them to get tested they didn't come”.*

Health staff also reported improved collaboration with community-based interventions which has helped bridge communication between services and PLHIV. In line with the LR approach HBC teams who had previously been responsible for the care and support of PLHIV expanded their scope of work to include referring all pregnant women to ANC for HIV testing, following up HIV-positive women and exposed infants, supporting PMTCT services, and conducting community awareness-raising. HBC representatives in OD1 appear to have embraced this expanded role. One member of staff at the OD1 “hub” detailed the benefits of this improved coordination as follows:

*“We communicate well… first of all hospital staff understand each other… we all know about our duties, and, on the other hand, we collaborate with partner organisations… we collaborate with Home Based Care teams and the community… If they (HBC teams) refer patients here, it means that they already explained things to them… On the other hand, when we have a problem such as someone not coming to get their test result… In the past we could only send a message, and no one responded. But now we send a message and we can receive their (the HBC team's) feedback”.*

HBC team FGD participants in OD1 also confirmed improved cooperation with health services as an important outcome of the LR, citing their role of providing PMTCT advice and follow-up to HIV-positive clients both at health posts and within their communities. They emphasised the importance of regular monthly meetings with OD-level health workers to discuss and resolve any difficulties. They also reported that their combined efforts with health staff to facilitate HIV testing during pregnancy had improved the overall functioning of maternal health service provision, by ensuring pregnant women were aware of both the importance and availability of HIV testing and testing was systematically integrated within other services. In the words of one HBC team member:

*“We contact the health centre and give counselling to them (pregnant women) a day before we make an appointment with them to draw their blood. If they have an appointment for vaccination and ANC check-up, we make an appointment for an HIV test for the same day”.*

Three of the eight PLHIV respondents from OD1 tested HIV-positive through the ANC testing system since the LR was operational; one through village outreach testing, one through a rural “Linked” health centre, and one at the “hub” (referral hospital). These findings correspond with routine data, which reveal that about a half of women enrolled in the LR were already known to be living with HIV and were referred through existing care networks [18]. The husbands of all eight respondents had also been tested; four were positive and four were HIV-negative (in two of the latter cases the couple had separated). All respondents had received ART medication for PMTCT during their pregnancy. Seven had delivered live newborns and one delivered a stillborn. The seven women with young babies were at various stages of having these infants tested for HIV.

All eight respondents reported accepting regular visits from HBC teams. Many explicitly described how the monthly visits involved a range of PMTCT support, including a discussion about ART treatment, with the HBC team encouraging drug adherence and taking newborns to be tested and advising on contraception (and in some instances distributing condoms), as well as more traditional HBC support. In the words of one woman:

*“The NGO… visits once a month… they ask questions about my health… whether we are well or not, whether I take medicine regularly or not. I tell them that I take medicine every day. The HBC team also advises on the prevention of HIV transmission, including condom use”.*

Other respondents detailed:

*“They (HBC teams) come to ask about our CD 4 level, is it increasing? And about our adherence to treatment and whether we use condoms…”*

*“He (HBC team member) tells me not to be frightened, to take my child to be tested for HIV… I feel happy… seeing them take care of me”.*

These findings confirm the vital contribution which HBC teams can play in reducing mortality through PMTCT follow-up as part of integrated RH service provision, an issue which has been poorly examined to date [12, 13].

All of the female PLHIV interviewed in OD1 had disclosed to their husband and family; indeed often husbands were reported as playing a vital role in treatment adherence. As one respondent described:

*“He (my husband) reminds me to take the medicine every day—he's afraid I won't take the medicine. And the book, the book for receiving medicine, I can't read, so I ask him to read it for me”.*

Similarly, in another IDI one husband reported:

*“I help her (my wife) go to meetings with the doctor on an appointment date… sometimes she forgets to take her drugs, when I remind her, she goes to take them”.*

Indeed, the female PLHIV interviewed in OD1 reported that husbands, mothers, and children were all playing a crucial role in keeping track of the two treatment doses to be taken each day, encouraging, and reminding the women to adhere to their ART regime. Families were also found to be complementing the follow-up work carried out by HBC teams (and vice versa). None of the PLHIV interviewed in OD1 expressed any problems in accepting regular visits from HBC teams. As one interviewee highlighted:

*“I feel normal when they (the NGO) visit as I am not afraid that my neighbours will know, or that people will know about our family as we have this disease”.*

Health staff from OD1 who participated in FGDs observed that the problem of discrimination of PLHIV within the local communities had decreased over time, and attributed this to awareness-raising activities by HBC teams conducted through their work as part of the LR. Similarly, HBC team members reported that over recent years stigmatization had become less prevalent. The role of HBC teams in educating neighbours/communities about HIV was also emphasised by a number of the HIV-positive women interviewed; some respondents made a direct correlation between such activities and a reduction in the stigmatization they experienced locally. In the words of one woman:

*“I informed other people about my status. My neighbours did not discriminate against me… The NGO provided education on transmission to all the people in the community so they no longer discriminate”.*

Such findings were markedly different from those from OD2. 

### 3.2. Service Provision and PLHIV Engagement with Services in OD without Integrated Approach

Of the two PLHIV respondents in OD2, one woman tested in around 2000 in the Cambodian capital Phnom Penh and one tested at the OD referral hospital in early 2010. In the first case the HIV status of the husband was not clear and the couple had separated when the respondent was pregnant with her most recent child; in the second case the husband was HIV-negative. One respondent had taken ART during her pregnancy and reported that she was planning on bringing her baby for follow-up HIV testing in the coming month; the other was three months pregnant at the time of interview and reported that she was “waiting” to be put on an ART regime.

Both HBC and health staff FGD participants in OD2 reported problems in identifying new HIV cases. One HBC representative expressed the hope that this would be resolved through the introduction of more comprehensive testing facilities as part of the LR:

*“In the past, we faced problems. We only have VCT in seven health centers (HCs), other HCs have pregnant women… but not every HC offers testing, so we have to refer them (pregnant women) to our partnership HCs… The national policy states that all pregnant women should have an HIV test, but now, this is easy as we are starting to implement LR in this district… blood can be drawn at all HCs”.*

At the time of the study HBC team representatives in OD2 reported that they were mainly working with preexisting cases—individuals requiring OI support and ART treatment, for example. The AIDS Coordinator interviewed in OD2 emphasised the poor counselling skills of health staff, which affected their ability to persuade pregnant women to be tested for HIV, attributing this to limited training. However, the low number of HIV-positive women recruited for PMTCT in OD2 may not only be related to poor testing rates, but also poor follow-up of women within the community; HBC representatives who participated in an FGD in this OD also highlighted low rates of collection of test results. In addition, it was reported that poor community understanding of HIV, coupled with discrimination against PLHIV were significant problems in this OD which may have been negatively influencing voluntary HIV testing amongst the general population as well as disclosure amongst PLHIV. One HBC representative detailed:

*“Up to the present time, there are still some patients that did not want to disclose their HIV status, it is a problem, even though we work with the community because sometimes we cannot go to their home… They do not want their neighbours to know about their HIV status, they do not even want their parents to know their status… One pregnant woman is undisclosed and does not want her mother to know and wants to deliver her baby without awareness of her HIV status… she agreed that when I distribute some food or other materials, she prefers to come to take the food, rice and other materials here”.*

Fear of discrimination as a barrier to engagement with service providers was confirmed by the two IDIs conducted with PLHIV in OD2. One explained her refusal of HBC as follows:

* “I did not want the outreach team to visit, this will make people know my status. And then the neighbours will talk. That's why I do not want them to visit me… to avoid my story becoming public”. *

The role of HBC teams in supporting PLHIV and their families, as well as their wider PMTCT and RH work within the remit of the LR, which was about to commence at the time of the study, was therefore severely compromised in this OD due to fear of disclosure amongst PLHIV. 

In contrast, experiences reported from OD1 appear to suggest that the more widespread introduction of HIV testing and integrated support improved the lives of PLHIV and their communities, including through decreasing stigmatization which enabled local teams to work more effectively: a cyclic impact. 

### 3.3. Factors Contributing to a Reduction in Stigma

At the same time, recent research suggests that stigmatization of PLHIV has declined over time in Cambodia on a national level [17], hence the reduction in discrimination observed by study participants in OD1 clearly cannot be attributed solely to the work of the LR and specific HBC teams. Notably, several respondents in OD1 highlighted the combined roles of various interventions in reducing myths about HIV transmission and hence stigmatization. One interviewee described, for example:

*“I told (disclosed to) my neighbours who live near my house… they told other people, so they all knew… They acted differently from before… They discriminated against us by rarely visiting… but after the organisation advised us all and from listening to the radio, they knew HIV/AIDS cannot be passed from one person to another by eating together… Now they are fine… we can eat together… ”*

It therefore appears that the increasing visibility of HIV within local communities, its perceived “normality” (as one PLHIV interviewee in OD1, diagnosed in 2003 described “… at that time many people discriminated against those with HIV. Now I feel normal, because there is no discrimination, we are normal like other people”) and “manageability” through the provision of ART and health services, and media education campaigns coupled with HBC team community awareness-raising work are all likely to have contributed to the low levels of reported stigmatization of PLHIV in OD1. Previous research in other regions has shown the positive impact which the support of community-based teams (or being a member of an association of people living with HIV and AIDS) might have on PLHIV and their children [19], including through the countering of stigmatization through community-based support teams [13, 20].

### 3.4. User Attendance of and Satisfaction with Integrated Reproductive Health/PMTCT Services

The eight female PLHIV interviewed in OD1, as well as their partners/family members, reported overall satisfaction with the medical services available to them through the LR. In all but one case these respondents had attended ANC regularly throughout their most recent pregnancy. Several women had attended ANC on a monthly basis, reporting that they were advised to do by medical staff. The exception was one woman who only attended ANC once when she was six months' pregnant (when she tested for HIV and found to be positive).

FGD participants in OD1 (not known PLHIV) who had delivered over the past year expressed the view that regular ANC attendance amongst local women was nowadays common and had increased since 2008. However, there was an indication that overall ANC attendance may have increased amongst pregnant women over recent years in *both* districts included in the study. Indeed, all FGD participants in both OD1 and OD2 reported having attended ANC regularly during their most recent pregnancy, an anomaly highlighted through an earlier analysis of routine data from ODs with LR and those without LR [18]. Further, all FGD participants in both OD1 and OD2 reported having tested for HIV during their most recent pregnancy. Such findings emphasise the importance of examining wider contextual factors beyond new service integration strategies which may also contribute to positive improvements in service uptake.

There was a consensus amongst all female PLHIV study participants that the system of service delivery was clear, and that medical staff were knowledgeable and impart clear information and advice. One interviewee spoke of the support she received from a health professional at the “hub” who was also HIV-positive:

*“One of the people junior to the doctor is also a (HIV) positive patient. They said ‘I am the same as you, so you don't need to worry about people discriminating against you' … If we can't read, they will help to explain about appointment dates and what time we have to get tested. They explained all of it”. *

None of the HIV-positive respondents in OD1 reported any problems in understanding the drug regime they were on or the PMTCT drugs given to their baby (although, as already noted, many rely on family members to help them remember when to take their medicine). There was a general consensus amongst these PLHIV that delivery services were sufficient and that HIV-positive women were treated “normally”, although descriptions of staff completely covered in protective gear suggested a highly cautious attitude amongst service providers. At the same time, there is also a very real danger of HIV transmission to doctors and midwives involved in delivery. The HIV/AIDS Coordinator in OD1 cited a case of amniotic fluid squirting into the eye of maternal health staff during one delivery, for example; this individual had to take preventive medicine. As is reported in detail below, some discriminatory behavior amongst certain medical staff was commented upon by a number of women PLHIV interviewed in OD1, although these users appeared to distinguish this treatment from the technical quality of the service they received.

### 3.5. Challenges in Effective Service Provision

Despite the fact that delivery is offered at “linked” health centres, “satellites,” and the “hub” as part of the LR in OD1, only one respondent reported delivering outside the “hub”. In most cases respondents described being advised by medical staff to deliver at the district referral hospital as this was considered to be the best equipped, including for emergency cases. One respondent originally intended to deliver at a “satellite” but was referred to the “hub” due to bleeding, resulting in a caesarian section. Hence lower level health centres appear to be underutilized for delivery by HIV-positive women. A lack of confidence in and possible fear of conducting deliveries for PLHIV amongst some staff—attitudes which can have a serious impact on HIV-positive service users—also became evident through women's descriptions of their delivery experiences. One respondent described how during her arrival at the “hub” for delivery the midwives were too nervous to proceed and waited for the “AIDS staff” to arrive. The baby was delivered stillborn and it is unclear whether the procedural delay affected this outcome. The woman in question expressed her anger:

*“They (the health staff) spoke like they blame me: you have already got HIV and you still want to have a baby. They spoke like they did not want to deliver, and were afraid of being infected… and they do not want to deliver for you… they discriminate against PLHIV although they know how HIV is transmitted, and how to prevent transmission… They are still afraid of transmission… After Mrs X (specialized HIV staff) came, she said please don't be angry… she did the delivery for me, she did suture for me, and she was not afraid like those midwives… she was modest, not aggressive… like the young midwives”.*

A second respondent reported similarly poor treatment:

*“When I arrived the doctors there (OD1 “hub”) were mean… They said that I am a HIV positive patient: even if they wear ten pairs of gloves; they still don't want to examine me. When they stitched my vagina, they didn't give me an injection. And it was my first child; they also not give me any healing injection or painkiller injection”.*

In addition to the detailed examples cited above, other PLHIV respondents criticised the attitude of some health staff towards them as stigmatizing, in contrast to the staff responsible for HIV test counselling, who they praised for their encouraging attitude. One respondent reported comments made to her by “hub” staff concerning her previous “easy” life focusing on “bed affairs” (presumably an allusion to sex work) when she was collecting her ART. Indeed, numerous reported interactions with “hub” staff in OD1 suggested that inappropriate attitudes were widespread. One women described how when she revealed her intention to deliver at the hospital this resulted in the joke “so you are coming here to give us HIV?!”. Another interviewee reported the advice she receives when she collects her ART drugs as follows:

*“Researcher: When you went to get drugs, did you receive a clear explanation… what did the health staff explain to you?*

*Respondent: They explained how to take care in order to have good hygiene, not be dirty, not make a bad smell when we sit next to other people… As people may say things like “she has AIDS and she has a bad smell… They advised me to keep taking the drugs… When I come to collect the drugs, I have to clean my teeth to prevent a smell of bad breath for the health staff, and to cover my mouth with a mask”.*

Further, several PLHIV reported comments from health staff about their fertility choice, both during ANC consultations and when presenting for delivery (“you already have HIV and you still want to have a baby?”). A certain level of acceptance of such treatment was apparent amongst those who reported it, which may be associated with the current lack of recourse. 

Such findings suggest that the previous separation of responsibility for HIV and other RH services may have led to a “ghettoization” of HIV-focused support, with those not used to working in this field not only feeling fearful of working with PLHIV but also having poor awareness of the human and fertility rights of PLHIV. This confirms findings elsewhere that the fertility/reproductive rights of people with HIV depend on eradicating discrimination among health professionals as well as amongst the wider community [21] and that discrimination may be more widespread amongst staff not specializing in HIV [22].

Reported experiences of women FGD participants (not identified as HIV-positive) who had attended ANC and delivery services in OD1 over a similar period as PLHIV interviewees were mixed. Some emphasised the patient nature of hospital ANC staff, while others voiced concerns about the attitude of certain “hub” staff. One young doctor in particular was described as rude in his manner and some FGD participants reported that certain health professionals are physically rough during ANC examinations. In her description of her discontent one FGD participant revealed a problem which is likely to face all women: the navigation of unfamiliar services:

*“No, I'm not satisfied (with the delivery service at the hospital)… Because they are rude… Some staff aren't friendly to patients when we arrive. They tell patients to “go in first”, but we don't know where to go because we've never been there before… They keep telling us to go in”.*

When interviewed, the HIV/AIDS Coordinator in OD1 acknowledged that the attitude of some staff under his management was problematic, highlighting how staff are often tired and overstretched. Similarly some hospital staff FGD participants mentioned their extreme tiredness which affected their attitude to clients. One individual who had been working at the OD1 referral hospital for more than five years described:

*“I think that sometime the doctors speak in formal language… that's why the patients think they are unfriendly. Or sometime the doctors are in a hurry and can't talk much… Some days there is so much work to do, so how can we keep smiling for a whole day? sometimes we don't even have time to drink a drop of water for a whole morning”.*

Some OD1 staff commented on the extra workload associated with the LR programme, and in particular their obligation to “accompany” new clients to the various services within the hospital, which takes time: one member of staff detailed:

*“Sometimes when they leave from the OI place after we've examined them already, we've told them to get the medicine. Some patients ask where they can get the medicine. How can we let them ask other people where they can get the HIV/AIDS medicine? They don't dare to ask… It's difficult for us to spend our time to take them to get the medicine. The other patients are waiting for us for such a long time”. *

These findings emphasise the workload faced by service providers working in integrated services, which can result in overstretched staff communicating poorly with users, echoing earlier research in sub-Saharan Africa on the potential dangers of increased provider workload and an associated reduction in the quality of care [9, 11, 23]. Again, users' lack of knowledge of and confidence in navigating services is apparent from the second quotation, which suggests a need for an adaptation of current approaches to alleviate the additional burden on health professionals of guiding clients in their use of the range of integrated services.

While the HIV/AIDS Coordinator in OD1 reported the LR approach to be a success, he qualified this by highlighting the importance of extending HIV testing to the broader population, including male partners. The Coordinator suggested continuing outreach activities to enable general community-based HIV testing, rather than requiring individuals to come to health points, an approach also recommended by HBC team representatives who attended FGDs in OD1, to facilitate more general testing, particularly amongst remote communities. The vital issue of increasing male partner testing has been noted in other studies [24].

## 4. Conclusions

Investment in district health services which take into account both HIV-related and reproductive health needs can produce significant improvements [10]. While the provision of integrated services at operational district level through the Linked Response has been shown to have demonstrable success in service provision [18] the current study underscores some of the qualitative aspects of that success. In general terms of improved service provider coordination and collaboration and improved two-way referral between various health facility levels, the intervention has had positive impacts. The provision of HIV testing through facilities situated closer to communities was also a positive development. Further, the expanded role of HBC teams played a vital role in referral, follow-up, and the effective provision of PMTCT, complementing the support of families of PLHIV regarding treatment adherence, for example, and, in some cases, providing women with HIV who do not wish to become pregnant access to contraception.

The study therefore offers important new evidence concerning the role and contribution of CBOs in the provision of integrated PMTCT and reproductive health services while also highlighting the importance of interventions at various levels: bringing integrated services and skilled community workers closer to households and communities is shown to be a validated approach, with observable outcomes.

Echoing findings in earlier studies on the possible overburdening impacts of comprehensive RH service provision, both management and staff reported time and workload pressures associated with the newly integrated services. The introduction of trained “guides” to facilitate users in their navigation of the various hospital services might alleviate the burden on health professionals in their current role of assisting clients in their use of the range of integrated services. Pregnant women or mothers who have had positive experiences could also be involved as resource persons in the communities, helping to explain the services and how they function and encouraging other women to utilize the services on offer.

The reported discrimination towards female PLHIV by general maternal health staff at district level, despite the introduction of the Linked Response, confirms recommendations elsewhere that strategic management of the linking of sexual and reproductive health and HIV will be necessary for this approach to act as a modality of stigma reduction [2]. Exaggerated fears of HIV infection and misperceptions about HIV transmission may also lead to overcautious care or delay in taking care of PLHIV, as already reported from Vietnam [25]. Careful strategies should be devised to improve health staff's sensitivity in dealing with HIV-positive clients. In order to diminish current stigmatization resources need to be invested in enhancing all service providers' awareness and understanding of the rights of PLHIV and improving interpersonal skills. Training is also necessary to build confidence amongst all maternal health staff in supporting deliveries amongst PLHIV. More can be done to allay the fears of health staff through refresher trainings on risk of HIV exposure, recommended protection measures, and more general mass media campaigns on HIV transmission and prevention. The introduction of a system of providing recourse to users who feel they are maltreated would also be advisable.

In the district where PMTCT and RH services were not yet integrated considerable work is required on awareness-raising, through multiple channels, to encourage HIV testing and counter community stigmatization of PLHIV and, at the same time, enable HBC teams to fulfill their roles. Using mobile phones to communicate with PLHIV who choose to remain undisclosed may be a helpful interim measure amongst HBC teams commencing the integrated approach in communities where stigmatization is still a problem (this strategy could also be particularly useful for contact with PLHIV living in remote areas).

The integrated programme could also redefine its target group and reach out to the general population such as men and women of reproductive age or couples planning to marry. Positive findings of the current study regarding husbands' involvement and support to women's ART/PMTCT treatment adherence provide resonance to the argument that new strategies are required to ensure more comprehensive involvement in integrated RH services by both women and men. Such a “shared intervention” approach would enable HBC teams and service providers to work more effectively to increase HIV testing rates and enhance PMTCT and more general ART adherence.
